# Altered gut microbiota and its metabolites correlate with plasma cytokines in schizophrenia inpatients with aggression

**DOI:** 10.1186/s12888-022-04255-w

**Published:** 2022-09-27

**Authors:** Hongxin Deng, Lei He, Chong Wang, Teng Zhang, Hua Guo, Hongwei Zhang, Yanning Song, Bangtao Chen

**Affiliations:** 1 Department of Psychiatry, Zhumadian Psychiatric Hospital (The Second People’s Hospital of Zhumadian), Zhumadian, 463003 China; 2 Department of Dermatology, Chongqing University Three Gorges Hospital, School of Medicine, Chong University, Chongqing, 404000 China

**Keywords:** Schizophrenia, Aggression, Gut microbiota, Short-chain fatty acid, Neurotransmitter, Inflammation, Oxidative stress, Leaky gut, Correlation

## Abstract

**Background:**

The pathophysiological mechanisms of aggression are manifold and they may closely interconnect. Current study aimed to determine the gut microbiota and its metabolites, and clarify their correlations with inflammation, oxidation, leaky gut and clinical profiles underlying aggression in schizophrenia (ScZ).

**Methods:**

Serum and stool specimens from ScZ inpatients with (ScZ-Ag, 25 cases) and without aggression (NScZ-Ag, 25 cases) were collected. Systemic inflammation, oxidation and leaky gut biomarkers were determined by ELISA, gut microbiota by 16S rRNA sequencing, short-chain fatty acids (SCFAs) by gas chromatography-mass spectrometry analysis and neurotransmitters by liquid chromatograph mass spectrometry analysis.

**Results:**

Significantly higher systemic pro-inflammation, pro-oxidation and leaky gut biomarkers were observed in ScZ-Ag than NScZ-Ag group (all *P*<0.001). Compared to NScZ-Ag group, the alpha-diversity and evenness of fecal bacterial community were much lower, the abundance of fecal genera *Prevotella* was significantly increased, while that *Bacteroides*, *Faecalibacterium*, *Blautia*, *Bifidobacterium*,*Collinsella* and *Eubacterium_coprostanoligenes* were remarkably reduced in ScZ-Ag group (all corrected *P*<0.001). Meanwhile, 6 SCFAs and 6 neurotransmitters were much lower in ScZ-Ag group (all *P*<0.05). Finally, a few strongly positive or negative correlations among altered gut microbiota, SCFAs, systemic pro-inflammation, leaky gut, pro-oxidation and aggression severity were detected.

**Conclusions:**

These results demonstrate that pro-inflammation, pro-oxidation and leaky gut phenotypes relating to enteric dysbacteriosis and microbial SCFAs feature the aggression onset or severity in ScZ individuals.

## Background

Approximate 1% of the global populations, especially the individuals in the late adolescence or early adulthood, are affected by schizophrenia (ScZ). It is a heterogeneous psychiatric syndrome involving a broad spectrum of clinical symptoms including positive symptoms (delusions, hallucinations, etc.), negative symptoms (anhedonia, social withdrawal, poverty of thought, etc.), and cognitive dysfunction [1, 2]. Usually, recurrent episodes of acute psychosis alternating with periods of full or partial remission characterize the nature of chronicity, and which is partially attributed to that current available treatment modalities are only for symptoms mitigation rather than complete cure. Furthermore, higher incidence of disability or premature mortality, and insupportable psychosocial burdens are the matters of greater concern [3, 4].

Aggression covers a variety of acts involving verbal threat, physical assault, and homicide. It is often subdivided into premeditated and impulsive subtypes [5, 6]. Aggression is more inclined to be an entity independent of any mental illness. Mounting evidence has emerged indicate a strong association between ScZ and aggression. Specifically, ScZ may augment the propensity for aggression incidence about fourfold to sevenfold, thus bringing a greater challenge for both mental health services and public safety in aggression-affected individuals with ScZ [7]. Pathogenesis of aggression remains largely unknown and its manifold aspects is further complicated by comorbidities including ScZ. The underlying pathophysiological changes determine the clinical management strategy for aggression and concomitant diseases.

We previously provided evidence that the systemic pro-inflammation response featured onset or severity of aggression in ScZ, and that was possibly caused by leaky gut-induced bacterial translocation [8]. Many peripheral cytokines could cross blood-brain barrier thus precipitating changes in mood and behavior through hypothalamic–pituitary–adrenal axis [9–11]. Unquestionably, the pro-inflammation phenotype is often a synergistic effect of multiple causes. Of these identifiable aspects, chronic pro-oxidative stress was uncovered to contribute aggression onset in intermittent explosive disorder [12], but that has rarely been confirmed in ScZ patients. In addition, an expanding body of literatures showed that enteric dysbacteriosis and dysbiosis of intestinal flora metabolites including short-chain fatty acids (SCFAs) or neurotransmitters may be integral parts of psychiatric disorders’ pathophysiology by altering state of oxidative stress and inflammation [13, 14].

Taken together, we hypothesize that the previously verified systemic pro-inflammation phenotype in aggression-affected ScZ cases involves alterations to gut microbiota and its metabolites, leaky gut, and oxidative stress. However, the profiles of these variables and their interrelationships are poorly investigated in ScZ inpatients with aggression. In this regard, the main goal of our current study was to profile gut microbiota structure and microbiota-derived SCFAs or neurotransmitters in ScZ inpatients with and without aggression, and further determine their correlations with plasma cytokines including systemic inflammation biomarkers [C-reaction protein (CRP) and tumor necrosis factor-α (TNF-α)], leaky gut-related biomarkers [intestinal fatty acid-binding protein (I-FABP) and Claudin-3], and oxidative stress indices [8-hydroxy-20-deoxy-guanosine (8-OH-DG) and 8-isoprostane (8-ISO)]. By clarifying these scientific issues, a more convincing theory and molecular strategies for the clinical management of ScZ as well as further studies are expected.

## Materials and methods

### Study population

The study was conducted in inpatients with ScZ with or without aggression behaviors within 1 week prior to admission during November 2020 to November 2021 in the Second People’s Hospital of Zhumadian, a tertiary psychiatric hospital in Henan Province, China. It was approved by and carried out under the guidelines of the Ethics Committee of the Second People’s Hospital of Zhumadian, and written informed consent was obtained from all the inpatients or the guardians of inpatients (if the patients were unable to sign consent because of poor intelligence) at the time of recruitment. Modified overt aggression scale (MOAS) and positive and negative syndrome scale (PANSS) was used to characterize aggression behaviors and psychiatric symptoms, respectively [15, 16]. Inpatient with a total MOAS score of zero or only having a score of one or more for verbal aggression was classified into the non-aggressive (NScZ-Ag) group, and MOAS score of five or more into the aggressive (ScZ-Ag) group. Patients’ demographic and clinical characteristics were collected as previously described [8].

All the subjects were aged ≥18 years. At sample collection, all included inpatients were at least 2 weeks of antipsychotics discontinuation. Diagnosis of ScZ was made by two board-certified psychiatrists according to the 10th edition of the international classification of diseases (ICD-10) criteria for ScZ. Exclusion criteria included (a) aggression behaviors not within 1 week prior to admission; (b) pregnant or lactating women; (c) presence of any other psychoses including affective disorder or substance abuse; (d) comorbidity with severe somatic diseases or neurological diseases; (e)comorbidity with other medical conditions such as parenchymal organ-specific diseases, immune-related diseases, hematological diseases, metabolic diseases, gastrointestinal diseases, and any history of gastrointestinal surgeries; (f) smokers with smoking index > 400; (g) drinkers with daily ethanol intake ≥40 g for men (20 g for women) in past 5 years or ≥ 80 g in past 2 weeks; (h) use of systemic antibiotics, corticosteroids or any other immunosuppressive therapy and oral probiotics in recent 6 months.

### Fecal sample collection, DNA extraction, and sequencing

As our study previously described [17], approximately 5 g of fresh stool samples from each patient were collected in a sterile plastic cup and stored at − 80 °C immediately. A corresponding ELISA kit (#13732, MEIMIAN, Jiangsu, China) was used to detect fecal calprotectin protein, one of intestinal inflammation indicators. QIAamp DNA stool mini kit (Qiagen, Hilden, Germany) was used to extract bacterial DNA from 200 ± 20 mg of feces according to the manufacturer’s instructions and NanoDrop ND-1000 Spectrophotometer (Nucliber) to confirm the purity of extracted DNA (A260/280 ratio of 1.8), all extracted DNA samples were stored at − 80 °C until further analysis. To analyze bacterial community in feces, the amplification of V3-V4 region of the 16S rRNA gene was performed using polymerase chain reaction (PCR) with bacterial universal primers 341F (5′-CCTACGGGNGGCWGCAG-3′) and 806R (5′-GGACTACHVGGGTATCTAAT-3′). All PCR reactions were performed in triplicate using a 50-μl mixture containing 1 × Phanta® Flash Master Mix(#P520,Vazyme,China),10 μM primers and 100 ng of template DNA. The amplification conditions were 98 °C for 30 sec (1 cycle), followed by 98 °C for 10 sec, 56 °C for 5 sec, 72 °C for 5 sec (30 cycles) and 72 °C for 1 min (1 cycle). Amplicons were then recovered from 2% agarose gels and further purified using AxyPrep DNA Gel Extraction Kit (Axygen Biosciences, CA, USA) and quantified by QuantiFluor -ST(Promega, USA) according to corresponding protocols. Purified amplicons were pooled in equimolar and paired-end sequenced (2 X 300) on an Illumina MiSeq platform (Illumina, San Diego, USA) according to the instruction. The raw reads were deposited into the NCBI Sequence Read Archive (SRA) database (Accession Number: PRJNA839224).

### Bioinformatic analysis

As our study previously described [17], paired-end raw reads with overlap were merged to tags and tags were clustered to operational taxonomic units (OTUs) at 97% sequence similarity using UPARSE (version 7.1, http://drive5.com/uparse/). The taxonomy of each 16S rRNA gene sequence was analyzed using the RDP Classifier Algorithm (http://rdp.cme.msu.edu/) against the Silva (SSU128) 16S rRNA database, with a confidence threshold of 70%. QIIME and R packages (v3.2.0) from the free online Majorbio I-Sanger Cloud Platform (www.i-sanger.com) were applied to analyze sequencing data. Briefly, alpha diversity indices (Shannon index and Simpson index), evenness indices (simpsoneven and shannoneven index) and richness estimators (ACE index, sobs index and Chao 1 index) at OUT level or Genus level were calculated using Mothur v.1.30.2. At Genus level, beta diversity assessment using principal coordinates analysis (PCoA) was conducted using R package and analysis of similarities (ANOSIM) analysis using R-vegan. Statistically significant differences in the relative abundances of taxa were determined by Wilcoxon rank-sum test. Linear discriminant analysis (LDA) was used for assessing taxa responsible for the differences between groups. Correlation between gut bacteria and clinical characteristics, cytokines, fecal SCAFs or neurotransmitters was determined by pearson’s correlation analysis in the VGAM package. Before the analysis, the indicator with variance inflation factor (VIF) more than 10 will be removed as it is considered to influence subsequent correlation analysis due to the existence of multicollinearity between selected factors.

### Measurement of IgA-coating gut Bacteria

As described previously [8], fecal pellets were collected from participants. The supernatants containing fecal bacteria were collected and washed with 1 mL PBS containing 1% bovine serum albumin. After incubation with blocking buffer, the fecal homogenates were stained with PE-conjugated Anti-human IgA and Live/dead dye for 30 min on ice. 96-well plates were coated with 1 mg/mL anti-IgA at 4 °C overnight. Fecal samples were diluted 1/200. Usually a twofold serial dilution was made. Samples were incubated at room temperature for 2 h, and biotinylated anti-IgA was added. 1 h later, HRP-conjugated streptavidin was added. The plates were developed by using TMB substrate and analyzed at 450 nm according to the manufacturer’s instructions.

### Fecal SCFAs and neurotransmitters determination

The tests were conducted in Majorbio Corporation (Shanghai, China). Fecal SCFAs and neurotransmitters were extracted from 100 mg solid stool sample with methanol-contained buffer. All ultrasonic shaking and centrifugation steps were performed in a 4 °C condition. The contents of extracted SCFAs and neurotransmitters were determined with gas chromatography-mass spectrometry (GC-MS) analysis and liquid chromatograph mass spectrometer (LC-MS) analysis, respectively.

### Blood sampling and laboratory detection

Fasting peripheral blood samples were collected from all the subjects at 8:00 a.m. The protein levels of indicators assessed by enzyme-linked immunosorbent assay (ELISA) in this study involved systemic inflammation biomarkers CRP (#E007462, 3ABio, Shanghai, China) and TNF-α (#489204, Cayman, Michigan, USA); intestinal inflammation or leaky gut-related biomarkers I-FABP (#DFBP20, R&D Systems, Minnesota, USA) and Claudin-3 (#abx250611, Abbexa, Cambridge, UK)]; oxidative stress indices 8-OH-DG (#589320, Cayman Chemical, Michigan, USA) and 8-ISO (#516351, Cayman Chemical, Michigan, USA)]. Assays were performed according to the specifications of the manufacturer and the detection limits were in line with the instructions of the manufacturer. Each serum sample was measured in duplicate. All the plates were read by the I MarkTM Micro plate Reader (Bio-Rad, Hercules, California,United States).

### Statistical analysis

Statistical analysis of the data compiled in Excel databank was conducted using SPSS/PC software (Version 19.0 for Windows; SPSS Inc., China). Categorical and continuous variables were expressed as number (%) or mean (M) ± SD, respectively. No outliers in values of cytokines by inspection of related boxplots. For comparisons of demographic information and clinical characteristics at baseline between groups, Fisher’s exact Chi-square test or *student t* test were conducted. Relationships between bacterial metabolites and cytokines or MOAS were determined by partial correlation analysis controlling for gender, age, BMI, episodes with ScZ, course with ScZ, and PANSS. All the tests were two-sided. A *P* < 0.05 was accepted as the cutoff for statistical significance, and a false discovery rate (FDR) was considered for the correction of the *P* value when determining differences in alpha diversity and compositions of fecal microbiota between groups.

## Results

### Inflammation and oxidation assessment

As Table 1 summarized, cohorts of ScZ inpatients with and without aggression shared similarities in gender ratio, age, BMI, ScZ episodes, ScZ course, total PANSS score and stool IgA content (*P* > 0.05 for all the variables). Compared with NScZ-Ag group, ScZ inpatients with aggression had significantly higher average total MOAS score (1.56 ± 0.82 vs. 14.56 ± 6.78, *P* = 0.000). Regarding biomarkers of systemic inflammation (CRP and TNF-α), intestinal inflammation (stool calprotectin, serum Claudin-3 and I-FABP) and pro-oxidation (8-OH-DG and 8-ISO), statistically significant differences between the two groups were observed and their concentrations all dramatically increased in ScZ-Ag group (all *P* < 0.05). On partial correlation analysis controlling potential confounders, MOAS showed positive association with serum level of CRP (*R* = 0.463, *P* = 0.001), TNF-α (*R* = 0.796, *P* = 0.000), I-FABP (*R* = 0.717, *P* = 0.000), Claudin-3 (*R* = 0.498, *P* = 0.001), 8-OH-DG (*R* = 0.397, *P* = 0.007), 8-ISO (*R* = 0.337, *P* = 0.024) and fecal calprotectin (*R* = 0.401, *P* = 0.006); and also, positive associations were detected between serum 8-OH-DG or 8-ISO and systemic inflammation biomarkers ( all *R*>0, *P* < 0.05).

**Table 1:** Clinic characteristics of all inpatients at baseline

Items	ScZ-Ag (*n* = 25)	NScZ-Ag (*n* = 25)	*P* value
Male, n(%)	8 (32.00)	9 (36.00)	0.996
Age, mean (SD), years	33.52 (8.94)	32.88 (8.91)	0.801
BMI, mean (SD), kg/m^2^	24.53 (3.55)	22.91 (2.59)	0.071
No. of ScZ episodes, n (%)	5.72 (2.88)	4.52 (2.84)	0.144
ScZ course, mean (SD), years	7.82 (3.78)	6.68 (2.56)	0.491
Total PANSS score, mean (SD)	66.12 (8.26)	62.72 (10.14)	0.216
Total MOAS score, mean (SD)	14.56 (6.78)	1.56 (0.82)	**0.000**
TNF-α,pg/mL	181.92 (54.43)	38.91 (10.48)	**0.000**
CRP,ng/mL	10.25 (5.21)	4.93 (3.88)	**0.000**
Stool calprotectin, μg/g	162.03 (99.19)	110.49 (33.43)	**0.017**
Stool IgA, OD	211.80 (41.35)	185.60 (69.39)	0.111
I-FABP, pg/mL	83.08 (20.32)	26.29 (4.87)	**0.000**
Claudin-3, ng/mL	56.95 (17.72)	35.63 (8.27)	**0.000**
8-OH-DG, pg/mL	13.54 (6.23)	8.19 (3.72)	**0.000**
8-ISO, pg/mL	27.59 (9.98)	19.18 (5.72)	**0.000**

*BMI* body mass index, *ScZ-Ag* schizophrenia with aggression, *NScZ-Ag* schizophrenia without any aggression, *PANSS* positive and negative syndrome scale, *MOAS* modified overt aggression scale, Chi-square or *student t* test was used for statistical differences between groups

### Fecal bacterial diversity

**Fig. 1:**
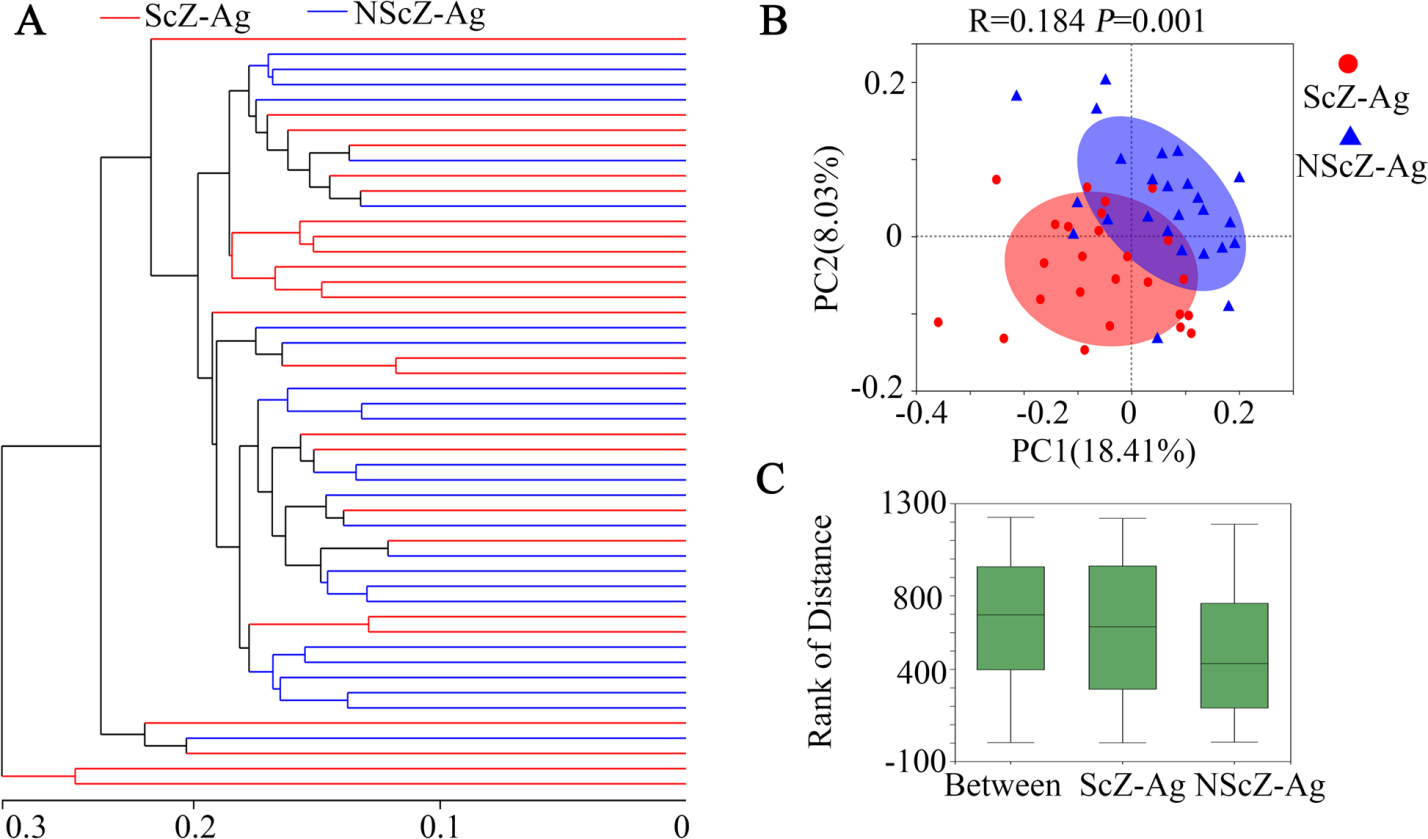
β diversity analysis of gut microbial structure. On Genus level, Hierarchical clustering tree (**A**), principal co-ordinates analysis (**B**) and comparison of distance rank by ANOSIM analysis (**C**) were performed as Methods specified. ScZ-Ag, schizophrenia with aggression; NScZ-Ag, schizophrenia without any aggression

From 25 fecal samples in ScZ-Ag group and 25 in NScZ-Ag group, a total of 1,257,221 valid sequences with mean length of 417 bp and 1,135,184 valid sequences with mean length of 419 bp were screened in the two groups, respectively. The reads from the two cohorts involved 1069 OTUs including 14 Phylum, 22 Classes, 51 Orders, 96 Families, 269 Genera and 565 Species. Clear asymptotes observed in refraction curve analyses with Sobs or Shannon index at Genus level indicate a near-complete sampling of the communities. As shown in Table 2, remarkably reduced values of index for ACE, Sobs or Chao at OUT level or Genus level in ScZ-Ag group demonstrating richness of fecal bacterial community in ScZ inpatients with aggression was significantly lower than that without (corrected *P*<0.01 for all variables). In comparison with NScZ-Ag group, Simpson estimator and values of index for Shannon, Shannoneven or Simpsoneven in ScZ-Ag group were remarkably higher and lower, respectively (corrected *P*<0.001 for all variables), which implies decreased alpha-diversity and evenness of fecal bacterial community in ScZ-Ag patients. At the Genus level, distinguished cohorts were visualized by Hierarchical clustering tree (Fig. 1A), furthermore, beta-diversity analyzed using the PCoA based on unweighted UniFrac metrics (Fig. 1B) and distance rank by ANOSIM analysis showed significant distinct clusters between the two cohorts (*R* = 0.184, *P* = 0.001, Fig. 1C).

**Table 2:** α diversity analysis in the two groups

Items	OUT level			Genus level		
	ScZ-Ag	NScZ-Ag	*P* value	ScZ-Ag	NScZ-Ag	*P* value
Sobs	220.56 ± 54.20	287.16 ± 58.14	0.000	88.40 ± 17.58	110.24 ± 21.57	**0.000**
Chao	282.15 ± 63.77	349.95 ± 75.84	0.002	101.28 ± 19.66	125.70 ± 28.40	**0.002**
Ace	284.48 ± 65.06	344.90 ± 68.62	0.003	101.82 ± 19.16	125.47 ± 25.57	**0.000**
Shannon	2.42 ± 0.53	3.36 ± 0.61	0.000	1.36 ± 044	2.48 ± 0.69	**0.000**
Simpson	0.20 ± 0.08	0.10 ± 0.08	0.000	0.52 ± 0.15	0.22 ± 0.16	**0.000**
Simpsoneven	0.03 ± 0.01	0.05 ± 0.03	0.000	0.02 ± 0.01	0.07 ± 0.04	**0.000**
Shannoneven	0.45 ± 0.08	0.60 ± 0.10	0.000	0.30 ± 0.09	0.53 ± 0.13	**0.000**
Good coverage	0.99 ± 0.00	0.99 ± 0.00	0.279	0.99 ± 0.00	0.99 ± 0.00	0.379
PD_ indexes	19.46 ± 3.89	23.51 ± 4.40	0.002	13.63 ± 2.43	15.94 ± 2.47	0.002

*OUT* operational taxonomic units, Wilcox rank sum test was used for statistical differences between groups

### Gut microbiota compositions and differences

**Fig. 2:**
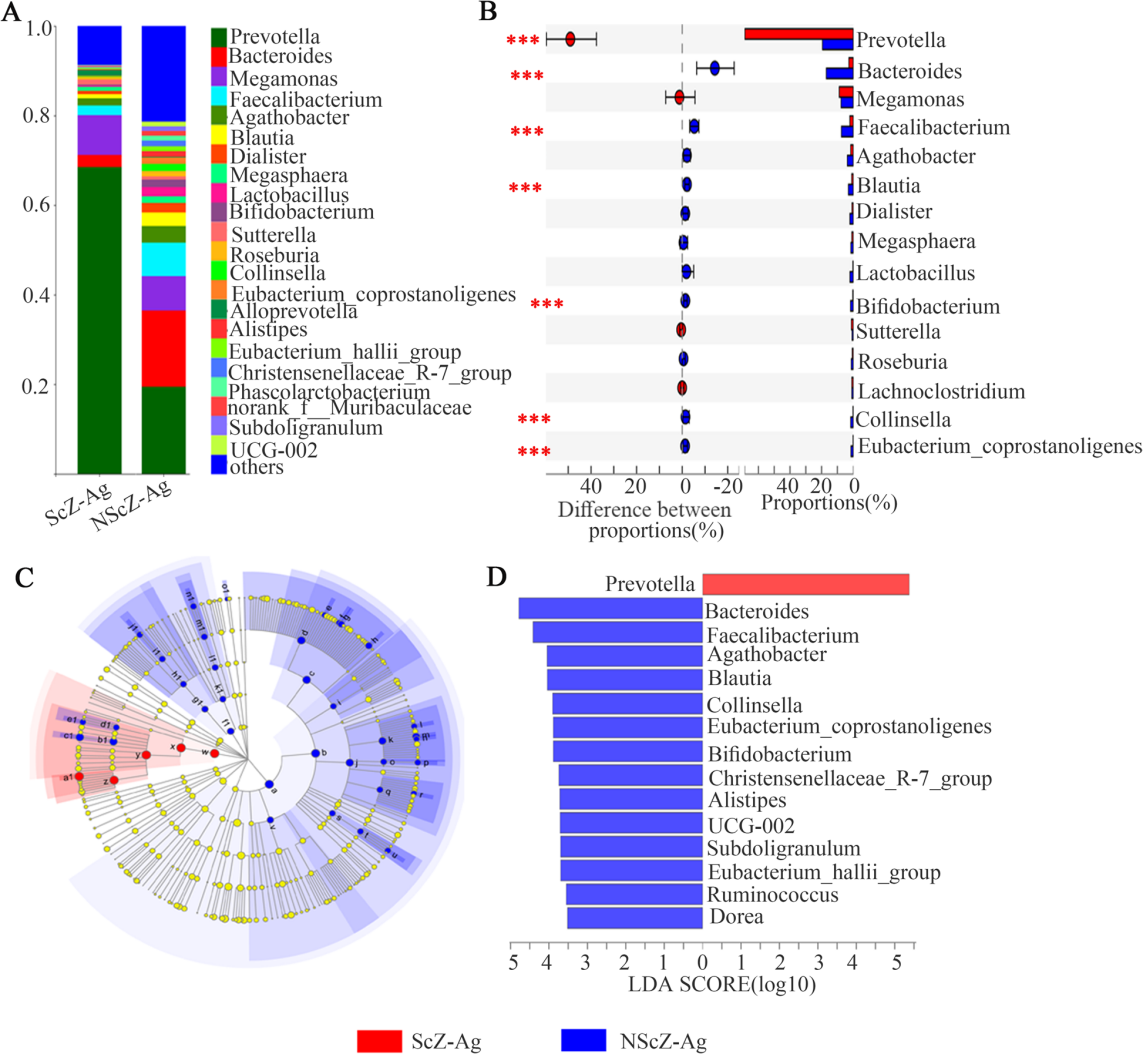
Analysis of fecal microbiota compositions and differences. The composition and relative proportion of gut bacteria in the two groups on Genus level (**A**). Wilcoxon rank-sum test bar plot on Genus level (**B**). Differently abundant taxa identified using LEfSe analysis from phylum to genus levels between the two groups (**C**). LDA showing the impact of different Genra on the difference between the two groups and visualization of only Genra meeting an LDA ≥3.5 (**D**)

There were 219 shared bacterium at the Genus level in both groups, with 13 and 37 unique ones in ScZ-Ag and NScZ-Ag group, respectively. More than 90% of fecal bacteria were Phyla *Bacteroidota* and *Firmicutes* in the two groups. Regarding the relative abundance at the Genus level (Fig. 2A), the top 5 taxa identified in ScZ-Ag group included: *Prevotella* (68.55%), *Megamonas* (8.88%), *Bacteroides* (2.69%), *Faecalibacterium* (2.15%) and *Agathobacter* (1.62%), and that in NScZ-Ag group were assigned to *Prevotella* (19.45%), *Bacteroides* (17.02%), *Faecalibacterium* (7.47%), *Megamonas* (7.18%) and *Agathobacter* (3.74%).

The relative abundance of Phyla *Bacteroidota* (73.41 ± 11.11 vs. 40.41 ± 18.53, corrected *P* = 0.000) in ScZ-Ag patients was markedly increased, while that *Firmicutes* (22.65 ± 11.31 vs. 51.56 ± 18.43, corrected *P* = 0.000) and *Actinobacteriota* (0.44 ± 0.49 vs. 3.51 ± 5.14, corrected *P* = 0.000) were remarkably decreased compared to NScZ-Ag controls. Differences in the relative abundances of top 15 genera composition of the intestinal microflora in the two groups were further analyzed using Wilcox rank sum test. Results showed that the ScZ-Ag group displayed a significant increase in *Prevotella* (68.52 ± 16.14 vs. 19.45 ± 24.11, corrected *P* = 0.000), while members of the *Bacteroides* (2.1 ± 2.48 vs. 17.36 ± 19.09, corrected *P* = 0.000)*, Faecalibacterium* (2.15 ± 2.09 vs. 7.46 ± 4.57, corrected *P* = 0.000), *Blautia* (0.92 ± 1.01 vs. 3.02 ± 2.17, corrected *P* = 0.000), *Bifidobacterium* (0.25 ± 0.42 vs. 1.67 ± 1.96, corrected *P* = 0.000), *Collinsella* (0.15 ± 0.15 vs. 1.67 ± 1.96, corrected *P* = 0.000) and *Eubacterium_coprostanoligenes* (0.13 ± 0.23 vs. 1.43 ± 2.21, corrected *P* = 0.000) were relatively decreased compared to NScZ-Ag group (Fig. 2B). Furthermore, application of LefSe method identified a total of 45 features with significantly different abundances between the two groups (LDA score > 3.5 with *P* < 0.05, Fig. 2C). Specifically, fecal microbiota of ScZ-Ag patients was differently enriched with genera *Prevotella* (*P* < 0.05), whereas the NScZ-Ag group was enriched with genera *Bacteroides, Faecalibacterium, Agathobacter, Blautia, Collinsella, Eubacterium_coprostanoligenes, Bifidobacterium, Christensenellaceae_R-7_group, Alistipes, Subdoligranulum, Eubacterium_hallii_group, Ruminococcus and Dorea* (*P* < 0.05 for all variables, Fig. 2D).

### Fecal SCFAs and neurotransmitters determination

Among various stool SCFAs except that similar levels of valeric acid and hexanoic acid, stool levels of acetic acid (125.09 ± 84.05 vs. 358.85 ± 75.31 ng/mg, *P* = 0.000), propanoic acid (141.68 ± 126.69 vs. 369.70 ± 151.45 ng/mg, *P* = 0.000), butyric acid (77.04 ± 52.51 vs. 335.93 ± 146.94 ng/mg, *P* = 0.000), isobutyric acid (12.18 ± 12.39 vs. 24.67 ± 20.19 ng/mg, *P* = 0.011), isovaleric acid (20.08 ± 30.51 vs. 70.85 ± 79.15 ng/mg, *P* = 0.004) and isohexanoic acid (0.12 ± 0.18 vs. 1.77 ± 3.01 ng/mg, *P* = 0.009) remarkably decreased in ScZ-Ag group compared to NScZ-Ag group (Table 3). Of the 23 fecal neurotransmitters determined, only 5-Hydroxytryptophan (0.04 ± 0.03 vs. 0.07 ± 0.03 μg/mg, *P* = 0.005), levodopa (0.17 ± 0.12 vs. 0.28 ± 0.24 μg/mg, *P* = 0.031), noradrenaline hydrochloride (8.48 ± 7.70 vs. 14.75 ± 12.20 μg/mg, *P* = 0.035), adrenaline hydrochloride (0.03 ± 0.04 vs. 0.07 ± 0.06 μg/mg, *P* = 0.009), kynurenic acid (0.08 ± 0.12 vs. 0.25 ± 0.23 μg/mg, *P* = 0.003) and histidine (0.73 ± 0.75 vs. 1.49 ± 1.91 μg/mg, *P* = 0.002) showed significant reductions at the concentration level in ScZ inpatients with aggression than those without (Table 4).

**Table 3:** Fecal SCFAs concentrations between the two groups

Items	ScZ-Ag	NScZ-Ag	*P* value
Acetic acid	125.09(84.05)	358.85(75.31)	**0.000**
Propanoic acid	141.68(126.69)	369.70(151.45)	**0.000**
Butyric acid	77.04(52.51)	335.93(146.94)	**0.000**
Isobutyric acid	12.18(12.39)	24.67(20.19)	**0.011**
Valeric acid	12.48(12.89)	19.27(15.66)	0.101
Isovaleric acid	20.08(30.51)	70.85(79.15)	**0.004**
Hexanoic acid	3.99(9.20)	10.09(18.65)	0.149
Isohexanoic acid	0.12(0.18)	1.77(3.01)	**0.009**

All the units for SCFAs are ng/mg and all data were presented as mean (SD). *Student t* test was used for difference analysis between ScZ-Ag and NScZ-Ag groups

**Table 4:** Fecal neurotransmitters concentrations between the two groups

Items	ScZ-Ag	NScZ-Ag	*P* value
Tryptamine	0.11(0.16)	0.17(0.66)	0.233
Tryptophan	12.56(10.73)	15.86(12.83)	0.329
Serotonin hydrochloride	1.13(2.64)	0.16(0.41)	0.351
5-Hydroxytryptophan	0.04(0.03)	0.07(0.03)	**0.005**
Dopamine hydrochloride	0.07(0.13)	0.12(0.20)	0.256
Levodopa	0.17(0.12)	0.28(0.24)	**0.031**
Noradrenaline hydrochloride	8.48(7.70)	14.75(12.20)	**0.035**
Adrenaline hydrochloride	0.03(0.04)	0.07(0.06)	**0.009**
Kynurenic acid	0.08(0.12)	0.25(0.23)	**0.003**
Kynurenine	0.10(0.11)	0.14(0.12)	0.292
Aminobutyric acid	13.67(12.79)	15.40(22.32)	0.737
Tyramine	12.25(13.48)	11.18(8.75)	0.741
Tyrosine	65.54(39.09)	73.10(39.28)	0.498
Histamine	5.11(6.82)	6.60(11.68)	0.452
Histidine	0.73(0.75)	1.49(1.91)	**0.002**
Glutamine	110.58(69.53)	121.02(89.04)	0.646
Glutamic acid	112.27(76.22)	124.14(103.27)	0.646
Picolinic acid	0.01(0.04)	0.02(0.07)	0.400
Acetylcholine chloride	2.62(3.41)	2.34(2.50)	0.738
5-Hydroxyindole-3-Acetic acid	0.09(0.11)	0.15(0.11)	0.073
Xanthurenic acid	1.73(1.93)	3.21(4.02)	0.105
Vanillymandelic Acid	0.68(1.22)	1.10(1.58)	0.298
Melatonine	0.001(0.001)	0.001(0.001)	1.000

All the units for neurotransmitter are μg/mg and all data were presented as mean (SD). *Student t* test was used for difference analysis between ScZ-Ag and NScZ-Ag groups

### Correlation analysis between variables

Finally, correlation analyses among intestinal microbiota, bacterial metabolites, cytokines and clinical characteristics were performed (Table 5). After controlling potential confounders as indicated, fecal concentration of acetic acid, butyric acid or propanoic acid was found to be strongly negatively correlated with serum TNF-α, I-FABP, Claudin-3 and MOAS (*R*<0, *P*<0.001 for all variables). In addition, there was a weak negative correlation 1) between CRP and acetic acid or propanoic acid, 2) between TNF-α and isobutyric acid, isovaleric acid, adrenaline hydrochloride or kynurenic acid, 3) between I-FABP and isovaleric acid, 5-Hydroxytryptophan, adrenaline hydrochloride, kynurenic acid or histidine (*R*<0, *P*<0.05 for all variables).

**Table 5:** Relations of bacterial metabolites to cytokines and MOAS

Variables	CRP	TNF-α	Calprotectin	I-FABP	Claudin-3	8-OH-DG	8-ISO	MOAS
Acetic acid	0.027*	0.000*	0.607	0.000*	0.000*	0.022*	0.112	0.000*
Propanoic acid	0.149	0.000*	0.717	0.000*	0.000*	0.380	0.475	0.000*
Butyric acid	0.023*	0.000*	0.099	0.000*	0.000*	0.004*	0.064	0.000*
Isobutyric acid	0.192	0.036*	0.120	0.055	0.342	0.407	0.492	0.017*
Isovaleric acid	0.206	0.046*	0.744	0.008*	0.081	0.240	0.721	0.054
Isohexanoic acid	0.826	0.141	0.246	0.168	0.309	0.250	0.224	0.132
5-Hydroxytryptophan	0.679	0.059	0.875	0.000*	0.000*	0.980	0.746	0.053
Levodopa	0.785	0.181	0.602	0.207	0.924	0.795	0.858	0.272
Noradrenaline hydrochloride	0.594	0.274	0.802	0.097	0.093	0.913	0.655	0.280
Adrenaline hydrochloride	0.802	0.031*	0.661	0.020*	0.115	0.060	0.393	0.051
Kynurenic acid	0.340	0.026*	0.470	0.016*	0.220	0.056	0.651	0.067
Histidine	0.102	0.099	0.781	0.018*	0.095	0.641	0.683	0.115

*P < 0.05. Analyses using partial correlation analysis. All the relevance indices (R)<0

**Fig. 3:**
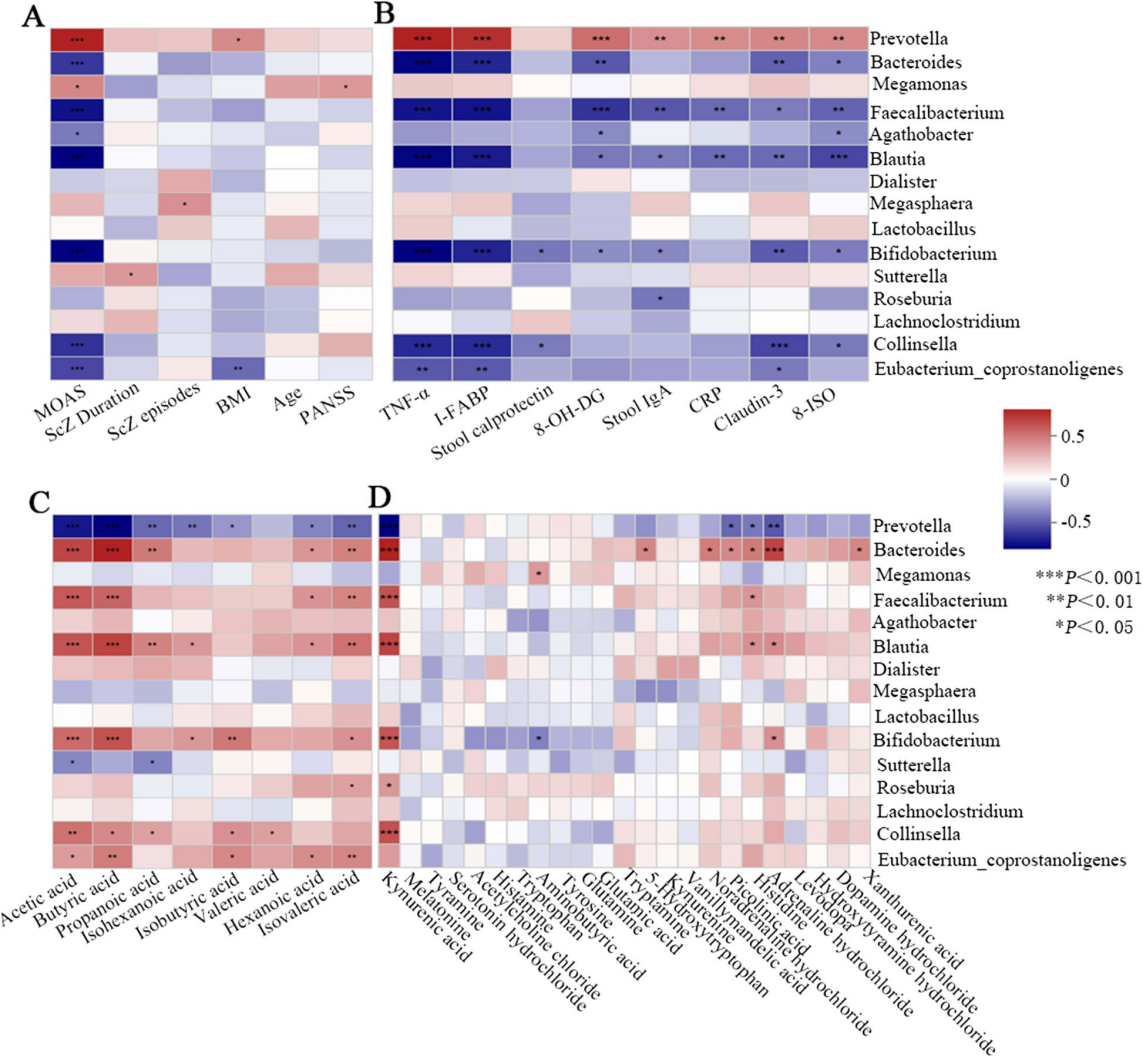
Correlation analysis of gut bacteria and clinical characteristics (**A**), cytokines (**B**), fecal SCFAs (**C**) or neurotransmitters (**D**)

As the correlation Heatmap graph showed (Fig. 3A), MOAS was strongly positively correlated with the abundance of the genera *Prevotella* (*R* = 0.628, *P*<0.001), whereas negatively with that *Blautia*, *Bifidobacterium*, *Faecalibacterium*, *Bacteroides*, *Collinsella* and *Eubacterium_coprostanoligenes* (*R*<0, *P*<0.001 for all variables). As Fig. 3B depicted, genera *Prevotella* showed strong positive correlation with TNF-α, I-FABP, and 8-OH-DG (*R*>0, *P*<0.001), and moderate with stool IgA, CRP, Claudin-3 and 8-ISO (*R*>0, *P*<0.01). Contrastly, TNF-α or I-FABP demonstrated a strong opposite correlation with genera *Bacteroides*, *Bifidobacterium, Faecalibacterium, Blautia and Collinsella* (*R*<0, *P*<0.001 for all variables)*.* Fecal acetic acid, butyric acid or kynurenic acid was also strongly oppositely linked with genera *Prevotella* (*R*<0, *P*<0.001 for all variables)*,* while positively with *Bacteroides*, *Bifidobacterium, Faecalibacterium and Blautia* (Fig. 3C&D, *R*>0, *P*<0.001 for all variables)*.* A moderate negative (*R* = -0.384, *P*<0.01) or strong positive (*R* = 0.508*, P*<0.001) association was found between fecal adrenaline hydrochloride and genera *Prevotella* or *Bacteroides,* respectively (Fig. 3D)*.*

## Discussion

Pathological aggression, one of the top 20 causes of disabilities, is a recognized global health concern that presents in about 33.3% (95%CI: 21.5% ~ 47.7%) of ScZ patients worldwide and in 15.3 ~ 53.2% of inpatients with ScZ in China [18, 19]. It often intersects with psychiatric disorders and thus adds to the complexity of treatment options. Clarifying the pathophysiological changes of aggression behaviors in specific comorbidity is a continuing need and efforts are in progress. Inflammation and oxidative stress disturbances are critical biological events in aggression pathogenesis in healthy or neuropsychiatric disorders including personality disturbance and intermittent explosive disorder [12, 20, 21]. We and other colleagues previously showed definitive evidence that systemic pro-inflammation phenotype characterizes aggression in ScZ cases [8]. However, state of oxidative stress and its correlation with inflammation response in aggression-affected ScZ individuals remain largely unknown. In current study, we found that ScZ inpatient with aggression had dramatically increased serum level of 8-OH-DG (nucleic acid oxidation biomarker) and 8-ISO (lipid oxidation biomarker) than those without, and further correlation analysis also showed positive correlativity between pro-oxidation and systemic pro-inflammation response or aggression severity. These results collectively suggest the co-contributory role of systemic pro-inflammation and pro-oxidation in the development of aggression in ScZ.

The factors that initiate or exacerbate pro-oxidation and systemic pro-inflammation in many conditions including neuropsychiatric disorders are being identified. Gut dysbacteriosis with leaky gut seems to play a vital role in the pathophysiology. Even though there are four animal-based studies revealing the link between aggressive behaviors and gut microbiota [22–25], only one patient-based clinical study preliminarily pointed to the possible correlation among aggression onset and gut dysbacteriosis [26]. In this study enrolling 42 ScZ patients (26 with violence and 16 without violence) without antipsychotics discontinuation before samples collecting, no significantly statistical difference in fecal microbiota richness, α- diversity and β-diversity were presented between the two groups [26]. In contrast, our data from ScZ inpatients with at least 2 weeks of antipsychotics discontinuation demonstrating ScZ inpatients with aggression had remarkably lower bacterial evenness, α- diversity, and β-diversity than those without. The inconsistent results can be partially attributed to the different living habits in different regions and antipsychotics discontinuation or not. Although the causal relationship remains controversial, decreased microbiota diversity and evenness underline the correlation between enteric dysbacteriosis and aggression etiology or severity in ScZ individuals, which further emphasizes the importance of subsequent in-depth analysis.

Difference in gut microbial taxonomic compositions is another consideration that associates with clinical events. Compared with controls, Chen et al. found that aggression-affected ScZ inpatients had a higher abundance of genera *Odoribacter*, and lower *Delftia* and *Rubrobacter* in feces [26]. However, relative increased abundance of genera *Prevotella,* whereas reduced abundance of genera *Bacteroides*, *Faecalibacterium*, *Blautia, Bifidobacterium, Collinsella and Eubacterium_coprostanoligenes* were detected in ScZ inpatients with aggression than the cases without in our study. For one thing, our result suggests that uncovering the variability of gut flora by sub-classifying the patients according to aggression presence or absence is warranted. For another, it also implies that correction of gut dysbiosis in aggression-affected ScZ inpatients using adjunct therapy with probiotics may achieve limited efficacy due to the variability in several bacteria besides Bifidobacterium, which is a verified speculation in some psychiatric disorders including ScZ [27, 28]. But from another point of view, emerging evidence confirmed the pro-inflammation properties of genera *Prevotella* [29–31], and anti-inflammation properties of genera *Bacteroides*, *Faecalibacterium*, *Blautia, Bifidobacterium* and *Collinsella* [31–34]. Biomarkers for aggression severity, systemic pro-inflammation, leaky gut and pro-oxidation showing moderate-to-strong positive correlation with the increased genus *Prevotella,* weak-to-strong negative correlation with the observed decreased genera collectively support the notion that enteric dysbacteriosis contributes to aggression by orchestrating the inflammation and oxidative stress responses.

Aggression is unanimously identified as one of the emotion recognition disorders. Brain and the intestine bidirectionally communicate (the so-called microbiota-gut-brain axis), and brain function modulation by gut microbiome occurs primarily through neuroimmune and neuroendocrine mechanisms [35]. Gut microbial metabolites including fecal SCFAs and various neurotransmitters intermediate in the complex bidirectional cross-talk system [36], but their variability in expression and relevance to clinical concerns in aggression occurrence are scarcely investigated. Herein, we found that SCFAs including acetic acid, propanoic acid, butyric acid, isobutyric acid, isovaleric acid and isohexanoic acid, and neurotransmitters including 5-Hydroxytryptophan, levodopa, noradrenaline hydrochloride, adrenaline hydrochloride, kynurenic acid and histidine remarkably decreased in ScZ cases with aggression than those without. Overall, a few positive or negative correlations between altered microbial metabolites and intestinal bacteria suggest that one metabolite can be produced by multiple gut bacteria and one bacterium may be involved in the productions of multiple metabolites, and furthermore, complex inter-regulatory interactions among bacteria may also existed, which are consistent with previous reports [37, 38]. SCFAs, especially the acetic acid, butyric acid or propanoic acid, are generally regarded to exert anti-inflammatory properties [39], which were further validated from our following correlation analyses. Moreover, severity of leaky gut (rather than intestinal inflammation), oxidation or aggression also showed opposite associations with the above mentioned SCFAs. However, of the 23 neurotransmitters, only fecal 5-Hydroxytryptophan demonstrated strongly negative with leaky gut, which may partially be attributed to the fact that the production and metabolism of intestinal neurotransmitters are more complex than those of SCFAs [40, 41]. These results further consolidate that gut microbiome contribute to pro-inflammation/oxidation-driven aggression in ScZ individuals through microbial metabolites, especially the SCFAs.

Limitations in current study include: 1) the causal relationships among specific enterobacteria, microbial metabolites, systemic inflammation, oxidative stress and specific aggression behaviors in ScZ patients to be inadequately explained as with all case-controlled clinical studies, related animal experiments are expected for ethical considerations. 2) within-subject verification of related biomarkers and replication procedures in larger study populations from multicenter are lacking, and a healthy control group in parallel is deficient. 3) the molecular mechanism by which enterobacteria and microbial metabolites promote inflammation or oxidative stress responses and thus drive aggression remains to be further investigated.

## Conclusions

The present study was the first to compare the state of inflammation, oxidation, intestinal microbiota and metabolites in ScZ inpatients with or without aggression. Results indicate pro-inflammation, pro-oxidation and leaky gut phenotypes relating to enteric dysbacteriosis and microbial SCFAs feature the aggression in ScZ individuals, which provides clues for future microbial-based or anti-inflammatory/oxidative therapies on aggression in ScZ cases.

## Data Availability

The raw reads of 16S rDNA sequencing were deposited into the NCBI Sequence Read Archive (SRA) database (Accession Number: PRJNA839224). Any other datasets generated during the current study are available upon reasonable request made to the corresponding author, without undue reservation.

## Abbreviations

ScZ: Schizophrenia
ScZ-Ag: ScZ inpatients with aggression
NScZ-Ag: ScZ inpatients without aggression
SCFAs: Sequencing, short-chain fatty acids
MOAS: Modified overt aggression scale
PANSS: Positive and negative syndrome scale
ICD: International classification of diseases
VIF: Variance inflation factor
LDA: Linear discriminant analysis
ANOSIM: Analysis of similarities
PCoA: Principal coordinates analysis
OTUs: Operational taxonomic units
GC-MS: Gas chromatography-mass spectrometry
LC-MS: Liquid chromatograph mass spectrometer
I-FABP: Intestinal fatty acid-binding protein
BMI: Body mass index
